# Atherosclerotic plaques occur in absence of intima-media thickening in both systemic sclerosis and systemic lupus erythematosus: a duplexsonography study of carotid and femoral arteries and follow-up for cardiovascular events

**DOI:** 10.1186/ar4489

**Published:** 2014-02-19

**Authors:** Marc Frerix, Johannes Stegbauer, Alexander Kreuter, Stefan Markus Weiner

**Affiliations:** 1 Department of Internal Medicine and Rheumatology, Justus-Liebig-University Giessen, Kerckhoff-Clinic Bad Nauheim, Benekestraße 2-8, Bad Nauheim, DE 61231, Germany; 2 Department of Internal Medicine and Nephrology, Düsseldorf University Hospital, Moorenstraße 5, Düsseldorf, DE 40225, Germany; 3 St Josef-Hospital Bochum, Department of Dermatology, University of Bochum, Venerology and Allergology, Gudrunstraße 56, Bochum, DE 44791, Germany; 4 Krankenhaus der Barmherzigen Brüder, Teaching Hospital of the University of Mainz, Department of Nephrology and Rheumatology, Nordallee 1, Trier, DE 54292, Germany

## Abstract

**Introduction:**

The objective of this cross-sectional and retrospective cohort study was (1) to determine the usefulness of intima-media thickness (IMT) in contrast to plaque assessment, (2) to examine the value of additive femoral artery sonography and (3) to identify potential risk factors for atherosclerosis and incident cardiovascular events in systemic sclerosis (SSc) and systemic lupus erythematosus (SLE) patients.

**Methods:**

In this study, 90 SSc and 100 SLE patients were examined by duplexsonography. IMT was measured in common carotid and common femoral arteries, plaques were assessed in common, internal and external carotid and common, proximal superficial and deep femoral arteries. Different definitions of pathological IMT (pIMT) were compared with the presence of plaque. Results were evaluated in relation to traditional and non-traditional risk factors for baseline atherosclerosis (logistic regression) and their predictive value for cardiovascular events during follow-up (cox regression).

**Results:**

Definite atherosclerosis occurred frequently without signs of subclinical atherosclerosis in both diseases: pIMT >0.9 mm was present in only 17/59 (28.9%) SSc and 13/49 (26.5%) SLE patients with already present atherosclerotic plaques. Using age-adjusted pIMT definitions, this rate was even lower (5.1-10.3% in SSc, 14.3-26.5% in SLE). Plaques were located only at the carotid or only at the femoral arteries in 26 (13.7%) and 24 (12.6%) patients, respectively. Age and nicotine pack-years were independently associated with atherosclerotic plaques in SLE and SSc patients, as well as the cumulative prednisolone dose in SSc subgroup, and ssDNA positive SLE patients had a lower risk for atherosclerotic plaque. During follow-up (available for 129/190 (67.9%) patients, 650 person-years), cardiovascular events occurred more often in patients with coronary heart disease (adjusted-hazards ratio (HR) 10.19, 95% confidence interval (CI) 3.04 to 34.17, *P* <0.001), male patients (adjusted-HR 8.78, 95% CI 2.73 to 28.19, *P* <0.001) and in patients with coexistent carotid and femoral plaques (adjusted-HR 5.92, 95% CI 1.55 to 22.67, *P* = 0.009). Patients with solely carotid or femoral plaque were not at higher risk.

**Conclusion:**

Atherosclerotic plaque lesions can be found frequently in absence of intima-media thickening in both SSc and SLE patients. As well as routine sonography of carotid arteries, the sonography of femoral arteries is recommended to identify additional atherosclerotic lesions and to detect patients at a high risk for cardiovascular events.

## Introduction

Several studies have reported different findings with respect to subclinical atherosclerosis (that is, pathologic thickened intima-media) and definite atherosclerotic lesions (that is, plaque) in connective tissue diseases [1-15].

Recently, two meta-analyses and reviews revealed a higher carotid intima-media thickness (cIMT) compared with healthy controls in both systemic sclerosis (SSc) patients and systemic lupus erythematosus (SLE) patients [1,2]. The majority of included studies limited duplexsonographic examination to the common carotid artery (CCA) for intima-media thickness (IMT) measurement. However, atherosclerotic plaques are often present at the section from the bulb to the internal carotid artery (ICA). Therefore, it is unclear whether several studies, which primarily focused on subclinical atherosclerosis, failed to detect already present atherosclerotic plaques.

Interestingly, in contrast to the results of the meta-analyses mentioned above, all large lupus studies in which atherosclerotic assessment was extended to segments distal to the CCA found a lower or a normal cIMT but a significantly higher rate of atherosclerotic plaques compared with age-matched and sex-matched controls [3-5]. Additionally, a decrease in small artery elasticity in SLE patients was observed without an increase of cIMT [6]. Hence, from these results atherosclerotic lesions seem to occur independently of subclinical atherosclerosis in SLE.

An increased prevalence of coronary heart disease in SSc patients was reported recently [7], but controversy remains as to whether accelerated atherosclerosis is a feature of SSc [8,9]. In one larger cohort of SSc patients, IMT or plaque frequency was not significantly different from controls [10], but whether the frequency of atherosclerotic plaque was underestimated by exclusion of ICA and external carotid artery (ECA) segments and an unusual strict definition of plaque is unclear. In contrast, another larger cohort revealed no difference in IMT but an increased prevalence of atherosclerotic plaques in carotid arteries (data published as abstracts only, details of IMT and plaque assessment unknown) [11,12]. Moreover, other available data from small SSc cohorts revealed conflicting results regarding cIMT and a low frequency of atherosclerotic plaques, but may be biased when ultrasound examination was limited to CCA [13,14]. In contrast, only in one available study extending the assessment of atherosclerotic plaques to all carotid artery segments (bilateral common, ICA and ECA) as well as vertebral arteries was a significantly higher rate of definite atherosclerotic lesions in SSc patients found [15].

Unknown state of premature atherosclerosis is of substantial interest for the affected patients, because Belcaro and colleagues previously showed in nonconnective tissue diseases that carotid and femoral artery lesions have a comparable power in predicting cardiovascular events (CVEs) [16]. Notably, the assessment of all four arteries predicted more events than scans of only carotid, only femoral arteries or only one side. There is only limited information on whether the femoral artery is affected by atherosclerosis in SLE [17], and studies searching for femoral artery plaques in SSc are lacking. CVEs were evaluated in several lupus cohort studies [18-20] and one SSc cohort study [21], but cohort studies including femoral artery duplexsonography are lacking.

Based on the potential impact of atherosclerosis on morbidity and mortality of SLE patients and SSc patients, our cross-sectional duplexsonography study of carotid and femoral arteries and retrospective cohort study of CVEs was designed to determine the usefulness of intima-media measurement in contrast to atherosclerotic plaque detection, to assess the value of femoral artery additive to carotid artery sonography for identification of patients at risk for incident CVEs, and to evaluate potential traditional and nontraditional risk factors associated with baseline atherosclerosis as well as their predictive value for incident CVEs during follow-up in both patient groups.

## Methods

### Patients

We retrospectively identified 90 consecutive SSc patients and 100 SLE patients from the inpatient and outpatient clinics of the Department of Dermatology, Venerology, and Allergology, University of Bochum, St Josef-Hospital Bochum and the Department of Nephrology and Rheumatology, Marienhospital Herne, University of Bochum between June 2004 and December 2006 to be eligible: SSc patients had to meet the criteria reported by LeRoy and Medsger [22] (and were subclassified into respective subsets: limited cutaneous SSc, *n* = 51; diffuse cutaneous SSc, *n* = 39) and SLE patients had to fulfill the American College of Rheumatology criteria [23]. Detailed characteristics of SSc and SLE patients are presented in Table 1.

**Table 1:** Baseline characteristics of systemic sclerosis and systemic lupus erythematosus patients

**Characteristic**	**Systemic sclerosis (*n*** **= 90)**	**Systemic lupus erythematosus (*n*** **= 100)**
Female	78 (86.7%)	87 (87%)
Age (years)	57.7 ± 14.4	48.1 ± 15.1
Disease duration (months)	85.0 ± 96.0	69.6 ± 92.0
Body mass index	24.5 ± 4.0	25.2 ± 4.7
Adipositas, body mass index >30	6 (6.7%)	14 (14%)
Hypertension	45 (50%)	56 (56%)
Systolic blood pressure (mmHg)	129.9 ± 23.2	130.6 ± 21.5
Diastolic blood pressure (mmHg)	75.9 ± 11.5	77.6 ± 10.6
Mean number of antihypertensives	0.8 (0, 4)	0.9 (0, 6)
Diabetes	4 (4.4%)	6 (6%)
Pulmonary hypertension	17 (18.9%)	3 (3%)
Coronary heart disease	10 (11.1%)	9 (9%)
Peripheral artery vessel disease	5 (5.6%)	2 (2%)
Nicotine use, current or former	43 (47.8%)	53 (53%)
Nicotine pack-years	10.7 ± 19.5	10.4 ± 16.2
Triglyceride (mg/dl)	139.2 ± 84.9	128.1 ± 69.9
Cholesterol (mg/dl)	216.5 ± 50.2	213.9 ± 59.6
Low density lipoprotein (mg/dl)	125.5 ± 39.2	123.7 ± 46.8
High density lipoprotein (mg/dl)	63.7 ± 18.7	64.4 ± 17.9
Family history of cardiovascular disease	12 (13.3%)	16 (16%)
Mean Rodnan skin score (median, range)	11 (9, 43)	–
Mean SLEDAI (median, range)	–	11 (9, 60)
Mean SLICC (median, range)	–	2 (2, 8)

Values are mean ± standard deviation, number (percentage), or median (range).

SLEDAI, Systemic Lupus Erythematosus Disease Activity Index; SLICC, Systemic Lupus International Collaborating Clinics damage index.

The study was conducted in accordance with the Declaration of Helsinki and guidelines of the local ethics committee of the Ruhr-University Bochum (separate ethics approval was not needed for this retrospective study according to the local ethics committee regulations). All data analyzed are based on routine clinical examination; no patient underwent additional examination procedure for the purpose of this study. Written informed consent was obtained from all patients for the use of clinical and duplexsonography data, and the use of blood samples for observational research purposes.

### Ultrasound examination

Baseline ultrasound examinations of carotid and femoral arteries of all 190 SLE and SSc patients were performed by two experienced investigators (JS and SMW) at the Department of Nephrology and Rheumatology, Marienhospital Herne, University of Bochum using the Siemens Acuson Sequoia 512 Ultrasound system (Siemens Medical Solutions, Erlangen, Germany) equipped with an 8 L5 linear transducer. Examinations were performed at 8.0 MHz for extracranial carotid and femoral arteries in several longitudinal and transversal section views to assess IMT and to identify atherosclerotic plaques (B-mode and color-coded duplexsonography). To avoid interobserver bias and because sonography examiners were not blinded for diagnosis in the routine clinical setting, the recorded anonymous images were evaluated only by one investigator (MF) without knowledge of patient data and characteristics.

### Carotid artery assessment of intima-media thickness and atherosclerotic plaques

We documented the mean of six IMT measurements at the far wall of the CCA over a 1 cm long segment, 1 to 2 cm proximal to carotid bifurcation for both sides. We compared the power of left and right IMT values and documented a mean IMT of the CCA as the mean of 12 measurements among both sides. The areas of plaque were not included in the IMT assessment. Because the term pathologic intima-media thickening (pIMT) for assessment in the CCA is widely used but not well defined, we compared three different definitions of pIMT: a European version (pIMT > 0.9 mm) [24], a German version (men 40 to 70 years old, pIMT > 1.0 mm; women 40 to 54 years old, pIMT > 0.85 mm and 55 to 70 years old, pIMT > 1.0 mm) [25], and an Atherosclerosis Risk in Communities (ARIC) version, defined by the 90th percentile for different age and sex groups out of the ARIC cohort [26]. To apply the most sensitive definition of pIMT, a patient was diagnosed to have pIMT of the CCA when the left or right IMT CCA fulfilled these conditions (even when the mean of left and right IMT CCA was lower). Additionally, a focal thickening of intima-media that did not fulfill the definition of plaque might have fulfilled the definition of pIMT.

We screened the arteries for carotid plaques in common, internal and external carotids. According to the Mannheim cIMT consensus, a plaque was defined as a focal thickening of intima-media >0.5 mm or 50% of surrounding intima-media into the arterial lumen, or a focal thickening >1.5 mm [27].

### Femoral artery assessment of intima-media thickness and atherosclerotic plaques

Analog to the assessment of mean IMT of the CCA, the mean IMT of the common femoral artery (CFA) was calculated as the mean of 12 IMT measurements at the far wall of the CFA (six at each side). Plaques were investigated with respect to the common, proximal superficial and deep femoral artery on both sides. Because femoral artery IMT measurement is not routinely performed, normal or rather pathologic IMT values as well as a definition of plaque are not given particularly for femoral arteries. Femoral artery plaque was thus defined similar to carotid artery plaque as a focal thickening of intima-media >0.5 mm or 50% of surrounding intima-media into the arterial lumen, or a focal thickening >1.5 mm [27]. In addition, we used and compared the definitions of pIMT and atherosclerotic plaque as given for carotid arteries with the findings at the femoral arteries.

### Assessment of traditional cardiovascular risk factors for atherosclerosis

The assessed traditional risk factors for atherosclerosis were age, sex, body mass index, arterial hypertension, diabetes mellitus, smoking (current or former and pack-years), dyslipidemia and family history of cardiovascular disease. Blood pressure levels were evaluated as the mean of left and right brachial artery measurements after at least 5 minutes of rest or (whenever possible) by 24-hour outpatient blood pressure measurement. Hypertension was defined by blood pressure >140/90 mmHg or use of antihypertensive drugs. In cases when a calcium channel blocker was prescribed for secondary Raynaud phenomenon, we did not rate this as antihypertensive drug use, except if arterial hypertension was documented previously and the calcium antagonist was given to treat hypertension and Raynaud phenomenon at the same time. Dyslipidemia was defined by elevated plasma cholesterol (>200 mg/dl for age 18 to 29 years; >220 mg/dl for age 30 to 40 years; >240 mg/dl for age >40 years), plasma low-density lipoprotein cholesterol (>160 mg/dl) and/or plasma triglyceride (>200 mg/dl) levels, or use of lipid-lowering drugs such as HMG co-inhibitors [28]. Plasma high-density lipoprotein cholesterol levels were assessed as a protective vascular factor.

### Assessment of potential nontraditional and disease-related determinants for atherosclerosis

We evaluated the following nontraditional and disease-related determinants as potential risk factors associated with accelerated atherosclerosis: in SLE patients, disease activity was assessed by the Systemic Lupus Erythematosus Disease Activity Index score (SLEDAI) [29] and disease damage was assessed by the Systemic Lupus International Collaborating Clinics damage index score (SLICC) [30]. In SSc patients we evaluated the modified Rodnan skin score [31]. Organ involvement was recorded including Raynaud phenomenon, pulmonary hypertension, coronary heart disease and peripheral arterial vascular disease (defined by obstructive vessel disease with claudicatio intermittens). We also assessed family history of connective tissue diseases, disease duration since diagnosis and age at diagnosis.

Previous immunosuppressive treatment was determined, such as use of prednisolone (duration of treatment, mean daily dose in the last 5 years and cumulative dose), hydroxychloroquine (HCQ), azathioprine (AZA) and cyclophosphamide (CYP) (duration of treatment and cumulative dose, if applicable). Sufficient data for duration of corticosteroid (CS) treatment were given in 96.8% of patients and for cumulative CS dose in 92.4% of patients. For duration of use and cumulative dose of HCQ, AZA and CYP, data were adequate in 99.5%, 98.4% and 99.5% of patients, respectively.

Routine laboratory assessment included C3c and C4c as disease activity markers for SLE patients, as well as C-reactive protein. Renal function was given by the glomerular filtration rate (Mayo formula). For analysis of the potential role of different autoantibodies, blood samples of all patients were tested by standard immunofluorescence test using Hep2 cells for antinuclear antibodies and by enzyme-linked immunosorbent assay (Euroimmune, Lübeck, Germany) for the following autoantibodies: double-stranded DNA (dsDNA), single-stranded DNA (ssDNA), Sm, C1q, SS-A, SS-B, U1RNP, Pm-Scl, RNP, RIP-PP, nucleosomes, centromeres, Scl-70 and antiphospholipid antibodies (cardiolipin IgG and IgM, β_1_-glycoprotein-1 IgG and IgM, lupus anticoagulans).

### Follow-up assessment of cardiovascular events

Patients were followed up by routine clinical visits at the Department of Dermatology, Venerology, and Allergology, University of Bochum. To compare the predictive value of pIMT, carotid and femoral artery plaques for CVEs, a review of patient records until December 2011 (5 years after baseline of the last included patient) was performed. CVEs were defined as coronary events (new angina pectoris or myocardial infarct requiring treatment), cerebrovascular events (transient ischemic attack or stroke), peripheral arterial vascular events (claudicatio intermittens, ischemic pain due to embolization) or death related to cardiovascular disease.

### Statistical methods

#### Baseline analysis of carotid and femoral artery duplexsonography assessment and potential risk factors for atherosclerosis (cross-sectional study)

Characteristics of SSc patients and SLE patients were described by univariate statistics. For comparison of mean IMT between the CCA and the CFA, we used a paired Student *t* test. The McNemar test was used to compare the frequency of carotid and femoral artery plaques. Different definitions of pathologic IMT were compared with the presence of atherosclerotic plaque in the whole cohort by descriptive statistics. Pearson correlation coefficients and phi coefficients are reported for correlation of potential risk factors for atherosclerosis with CCA and CFA IMT. For comparison of scale variables between patients with and without atherosclerotic plaque we used a two-sided Student *t* test, for non-normally distributed variables we used the Mann–Whitney *U* test. For comparison of categorical variables we used the chi-square test, or Fisher’s exact test if conditions were not confirmed.

Additionally, to assess factors independently associated with atherosclerosis, a multivariate linear regression analysis was performed for mean IMT of the CCA and the CFA, and a binary logistic regression analysis was performed for atherosclerotic plaque. Covariates for regression analyses were selected based on clinical knowledge and from potentially associated variables in explorative baseline analysis; model building was performed taking care of statistical considerations such as a suitable number of events per variable and the number of observations (observations with missing values were excluded from analyses). Competing models fit to the same set of data were compared using *R*^2^ measures (linear model), Nagelkerke’s pseudo-*R*^2^ and a likelihood ratio test (logistic model). The *R*^2^ value, constant, beta coefficients with 95% confidence intervals (CIs) and standardized beta coefficients were reported for the final chosen model of multivariate linear regression analysis; pseudo-R^2^ value, constant, beta coefficients and odds ratios with 95% CIs were stated for the final chosen model of binary logistic regression analysis. The goodness-of-fit of the logistic model was evaluated by Hosmer–Lemeshow test. To improve ease of interpretation of the continuous variables in the final logistic model (age, nicotine pack-years, prednisolone intake and AZA use), they were reported as 5-year increments.

#### Longitudinal analysis of cardiovascular events during the follow-up period (retrospective cohort study)

The risk of CVEs during follow-up for patients with presence of carotid and femoral artery plaques was compared with patients with only carotid or femoral artery plaque, as well as with patients without atherosclerotic plaques reported as rate ratio estimates with 95% CIs based on the incidence density rate (IDR; CVEs per person-years), using the Kaplan–Meier method to graph and the log-rank test to compare (unadjusted) survival curves for the time to first CVE. Additionally, the Cox proportional-hazards regression model was used to estimate unadjusted and adjusted hazard ratios (HRs) with 95% CIs for possible risk factors predictive of CVEs. Competing models were compared using the likelihood ratio test, and assumption of proportional hazards was confirmed by log-minus-log survival plots. Because the HR of patients with only carotid or femoral artery plaque and of patients without atherosclerotic plaque was nearly one, the polytomous variable for plaque (reported in Cox model 1) was dichotomized to build a parsimonious final "best" model (reported in Cox model 2). In the end, we built a Cox model using only the information of plaques at the level of carotid arteries to estimate the adjusted hazard of patients with carotid plaque in a hypothetical scenario in which screening of femoral arteries was not performed.

Statistical analysis was performed using PASW Statistics, Version 18 (SPSS Inc., Chicago, USA) and IBM SPSS Statistics, Version 21 (IBM, New York, USA).

## Results

### Comparison of baseline carotid and femoral artery duplexsonography results in systemic sclerosis patients

#### Comparison of carotid and femoral artery intima-media thickness in systemic sclerosis patients

IMT was highly correlated between left and right sides at the level of the CCA (*r* = 0.880; *P* < 0.001) as well as the CFA (*r* = 0.811; *P* < 0.001). The correlation between IMT of the CCA and IMT of the CFA was much weaker (*r* = 0.508; *P* < 0.001). There was no significant difference between mean CCA IMT and CFA IMT (0.76 ± 0.15 mm vs. 0.78 ± 0.18 mm; *P* = 0.170), or between left and right CCA IMT (0.75 ± 0.15 mm vs. 0.75 ± 0.16 mm; *P* = 0.707) or left and right CFA IMT (0.78 ± 0.19 mm vs. 0.77 ± 0.19 mm; *P* = 0.398).

#### Comparison of carotid and femoral artery definitive atherosclerosis (plaques) in systemic sclerosis patients

**Figure 1:**
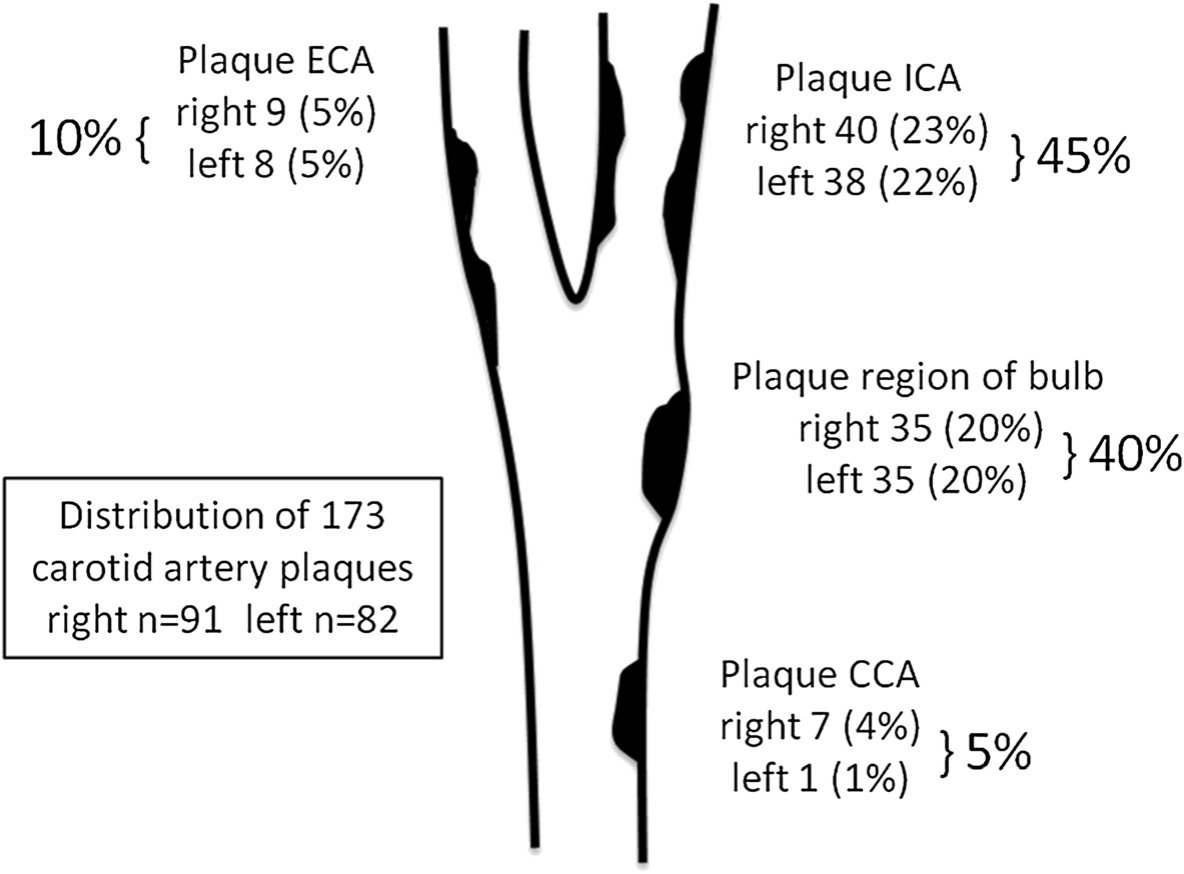
**Distribution of carotid artery plaques in our cohort of systemic sclerosis and systemic lupus erythematosus patients.** Carotid artery plaques are mostly located at the internal carotid artery (ICA) and the region of the bulb, and are rarely observed at the external carotid artery (ECA) and the common carotid artery (CCA). An extension of carotid ultrasound examination distal to the CCA segment for plaque assessment is highly recommended.

A total of 84 carotid artery plaques and 90 femoral artery plaques were detected in 59 SSc patients. Thirty-five carotid artery plaques were found in 27 patients (30%) on the left side compared with 49 plaques in 36 patients (40%) on the right side (*P* = 0.123). The distribution of carotid artery plaques according to the CCA, the region of the bulb, ICA and ECA is shown in Figure 1. In 9 to 18 patients (10 to 20%) carotid plaques would not have been detected if carotid ultrasound examination was limited to one side.

Forty-three femoral artery plaques were found in 36 patients (40%) on the left side and 47 plaques in 39 patients (43.3%) on the right side (*P* = 0.646). In 8 to 23 patients (8.9 to 25.6%) the plaques would have been missed if femoral ultrasound examination was limited to one side. Twenty-six patients (28.9%) had atherosclerotic plaques in only one of the four vascular territories. The overall frequency of carotid and femoral artery plaques was not different (50% vs. 52.2%; *P* = 0.845), but the plaque distribution varied in individual patients: in 12 patients (13.3%), plaque was only located at the carotid arteries; in 14 patients (15.6%), plaque was observed isolated at femoral arteries. In approximately every sixth to seventh patient, atherosclerosis would not have been detected if routine ultrasonography was limited to the carotid arteries.

#### Comparison of different pathologic intima-media definitions for common carotid artery with the presence of atherosclerotic plaque in systemic sclerosis patients

There was a high incongruity in the presence of atherosclerotic plaques and a thickened intima-media (pIMT), independent of the criteria used to define pIMT (Table 2). IMT > 0.9 mm was detected in only 17 out of 59 SSc patients (28.9%) with atherosclerotic plaques. Moreover, according to the European definition as well as the German definition, none of the patients without atherosclerotic plaques had a pathologic intima-media. Using the ARIC definition (taking note of individual difference in IMT according to sex and increasing IMT with age), 19.4% of SSc patients without atherosclerotic plaques had pIMT. Subgroup analysis revealed that about 80% of SSc patients with baseline cardiovascular disease had atherosclerotic plaques, in contrast to one-third of patients with pIMT (Table 3).

**Table 2:** Comparison of intima-media thickening with the presence of carotid or femoral atherosclerotic plaques (whole cohort)

**SSc and SLE patients with and without plaque**		**European definition (pIMT > 0.9 mm)**	**German definition (pIMT > 0.85/> 1.0 mm)**	**ARIC definition (pIMT > P90)**
SSc	Without plaque (*n* = 31)	0 (0%)	0 (0%)	6 (19.4%)
	With plaque (*n* = 59)	17 (28.9%)	3 (5.1%)	6 (10.3%)
SLE	Without plaque (*n* = 51)	0 (0%)	0 (0%)	3 (5.9%)
	With plaque (*n* = 49)	13 (26.5%)	7 (14.3%)	13 (26.5%)

Values presented as number of patients with pIMT (percentage).

pIMT, pathologic intima-media thickening; SLE, systemic lupus erythematosus; SSc, systemic sclerosis.

**Table 3:** Subgroup analysis of intima-media thickening and atherosclerotic plaques in systemic sclerosis and systemic lupus erythematosus patients with cardiovascular disease

**Subgroup of SSc and SLE patients with cardiovascular disease**		**Pathologic IMT (any definition)**	**Atherosclerotic plaque**			
			**Carotid and/or femoral artery plaque**	**Only carotid artery plaque**	**Only femoral artery plaque**	**Coexistent carotid and femoral artery plaque**
SSc patients with	Arterial hypertension (*n* = 45)	16 (35.6%)	38 (84.4%)	7 (15.6%)	5 (11.1%)	26 (57.8%)
	Pulmonary hypertension (*n* = 17)	6 (35.3%)	15 (88.2%)	0 (0%)	2 (11.8%)	13 (76.5%)
	Coronary heart disease (*n* = 10)	2 (20%)	8 (80%)	0 (0%)	0 (0%)	8 (80%)
	Peripheral artery vessel disease (*n* = 5)	2 (40%)	4 (80%)	1 (20%)	1 (20%)	2 (40%)
SLE patients with	Arterial hypertension (*n* = 56)	15 (26.8%)	39 (69.6%)	10 (17.9%)	9 (16.1%)	20 (35.7%)
	Pulmonary hypertension (*n* = 3)	1 (33.3%)	3 (100%)	1 (33.3%)	1 (33.3%)	1 (33.3%)
	Coronary heart disease (*n* = 9)	5 (55.6%)	9 (100%)	1 (11.1%)	0 (0%)	8 (88.9%)
	Peripheral artery vessel disease (*n* = 2)	1 (50%)	2 (100%)	0 (0%)	0 (0%)	2 (100%)

Values presented as number (percentage) of patients with pathologic intima-media thickness and carotid or femoral artery plaque.

IMT, intima-media thickness; SLE, systemic lupus erythematosus; SSc, systemic sclerosis.

### Association of potential risk factors with intima-media thickness and atherosclerotic plaques in systemic sclerosis patients

#### Correlation of traditional cardiovascular risk factors and disease-related factors with intima-media thickness in systemic sclerosis patients

Correlations between common carotid and femoral artery IMT with traditional and nontraditional risk factors for atherosclerosis are shown in Table 4 (and Additional file 1). In multivariate analysis, age was independently associated with IMT of the CCA and the CFA, whereas male sex was associated with IMT of the CCA and the CFA and decrease of renal function and smoking only with IMT of the CFA (Table 5).

**Table 4:** **Correlation of carotid and femoral artery intima-media thickness with traditional and nontraditional risk factors for atherosclerosis**^**a**^

**Variable**	**SSc (*n*** **= 90)**				**SLE (*n*** **= 100)**			
	**Mean CCA IMT**		**Mean CFA IMT**		**Mean CCA IMT**		**Mean CFA IMT**	
	**Correlation**	***P *** **value**	**Correlation**	***P *** **value**	**Correlation**	***P *** **value**	**Correlation**	***P *** **value**
Age (years)	*r* = 0.682	<0.001	*r* = 0.565	<0.001	*r* = 0.726	<0.001	*r* = 0.560	<0.001
Age at diagnosis (years)	*r* = 0.652	<0.001	*r* = 0.586	<0.001	*r* = 0.670	<0.001	*r* = 0.437	<0.001
Disease duration (months)	*r* = -0.030	0.782	*r* = -0.109	0.308	*r* = 0.094	0.357	*r* = 0.217	0.032
Male sex	*r*φ = 0.261	0.013	*r*φ =0.341	0.001	*r*φ = 0.268	0.007	*r*φ = 0.208	0.039
Postmenopausal status	*r* = 0.546	0.001	*r* = 0.375	0.001	*r* = 0.400	<0.001	*r* = 0.373	<0.001
Body mass index	*r* = 0.128	0.241	*r* = 0.197	0.070	*r* = 0.258	0.012	*r* = 0.294	0.004
Systolic blood pressure (mmHg)	*r* = 0.303	0.007	*r* = 0.332	0.003	*r* = 0.346	0.001	*r* = 0.171	0.124
Glomerular filtration rate	*r* = -0.316	0.003	*r* = -0.489	<0.001	*r* = -0.296	0.003	*r* = -0.277	0.006
Nicotine pack-years	*r* = 0.022	0.839	*r* = 0.320	0.002	*r* = 0.082	0.424	*r* = -0.058	0.570
Coronary heart disease	*r*φ = 0.160	0.133	*r*φ = 0.379	<0.001	*r*φ = 0.265	0.008	*r*φ = 0.160	0.115
mRSS	*r* = 0.225	0.036	*r* = -0.021	0.843	–	–	–	–
SCL70 antibody	*r*φ = -0.117	0.270	*r*φ = -0.209	0.048	–	–	–	–
Centromere antibody	*r*φ = 0.165	0.119	*r*φ = 0.255	0.015	–	–	–	–
SLEDAI	–	–	–	–	*r* = -0.166	0.115	*r* = -0.057	0.591
SLICC	–	–	–	–	*r* = 0.208	0.049	*r* = 0.117	0.271
dsDNA antibody	–	–	–	–	*r*φ = -0.188	0.063	*r*φ = -0.197	0.051
Nucleosomes antibody	–	–	–	–	*r*φ = -0.231	0.021	*r*φ = -0.123	0.224

CCA, common carotid artery; CFA, common femoral artery; dsDNA, double-stranded DNA; IMT, intima-media thickness; mRSS, modified Rodnan Skin Score; SLE, systemic lupus erythematosus; SLEDAI, Systemic Lupus Erythematosus Disease Activity Index; SLICC, Systemic Lupus International Collaborating Clinics damage index; SSc, systemic sclerosis.

^a^Only variables suggested for multivariate linear regression analysis are reported; complete report available in Additional file 1.

**Table 5:** Linear regression analysis of factors associated with carotid and femoral artery intima-media thickness in systemic sclerosis and systemic lupus erythematosus patients

**Variable**	**Mean CCA IMT**			**Mean CFA IMT**		
	**Standardized beta coefficient**	**Regression coefficient (95% CI)**	***P *** **value**	**Standardized beta coefficient**	**Regression coefficient (95% CI)**	***P *** **value**
**Systemic sclerosis**^**a**^	(Constant 0.349; *R*^2^ = 50.5%)			(Constant 0.762; *R*^2^ = 49.9%)		
Age (per year)	0.664	0.007 (0.005 to 0.008)	<0.001	0.336	0.004 (0.002 to 0.007)	0.003
Male sex	0.202	0.088 (0.022 to 0.153)	0.012	0.245	0.138 (0.025 to 0.251)	0.018
Glomerular filtration rate (per unit)	–	–	–	-0.302	-0.003 (0.000 to 0.004)	0.005
Nicotine use (per pack-year)	–	–	–	0.198	0.002 (0.000 to 0.004)	0.046
**Systemic lupus erythematosus**^**b**^	(Constant 0.392; *R*^2^ = 52.7%)			(Constant 0.452; *R*^2^ = 31.3%)		
Age (per year)	0.726	0.006 (0.005 to 0.008)	<0.001	0.560	0.005 (0.004 to 0.007)	<0.001

CCA, common carotid artery; CFA, common femoral artery; CI, confidence interval; IMT, intima-media thickness.

^a^Multivariate linear regression model for mean CCA IMT and CFA IMT of systemic sclerosis patients. ^b^Simple linear regression model, other additional variables in multivariate analysis did not significantly improve the model for both mean CCA IMT and CFA IMT of systemic lupus erythematosus patients.

#### Association of traditional cardiovascular and disease-related risk factors with presence of atherosclerotic plaques in systemic sclerosis patients

Detailed results of univariate analysis are presented in Table 6 (and Additional file 2). In multivariate analysis, age and cumulative nicotine pack-years were significant independent predictors for atherosclerosis and also cumulative prednisolone dose remained a statistically significant predictor of atherosclerotic plaques in SSc patients (Table 7).

**Table 6:** **Explorative baseline analysis of potential risk factors between patients with or without atherosclerotic plaques**^**a**^

**Characteristic**	**SSc (*n*** **= 90)**			**SLE (*n*** **= 100)**		
	**With plaque**	**Without plaque**	***P value***	**With plaque**	**Without plaque**	***P *** **value**
	**(*n*** **= 59)**	**(*n*** **= 31)**		**(*n*** **= 49)**	**(*n*** **= 51)**	
Age (years)	63.9 ± 12.0	45.9 ± 10.9	<0.001	57.7 ± 10.6	39.0 ± 13.0	<0.001
Male sex	10 (16.9%)	2 (6.5%)	0.206	11 (22.4%)	2 (3.9%)	0.007
Postmenopausal status, women only	44/49 (89.8%)	12/29 (41.4%)	<0.001	31/38 (81.6%)	16/49 (32.7%)	<0.001
Hypertension	38 (64.4%)	7 (22.6%)	<0.001	39 (79.6%)	17 (33.3%)	<0.001
Nicotine pack-years	13.9 ± 22.5	4.1 ± 7.8	0.003	14.7 ± 20.6	6.2 ± 8.9	0.009
Dyslipidemia	30 (50.8%)	7 (22.6%)	0.010	24 (49%)	15 (29.4%)	0.045
Age at diagnosis (years)	56.0 ± 14.3	38.7 ± 10.9	<0.001	50.8 ± 14.4	33.4 ± 11.4	<0.001
Pulmonary hypertension	15 (25.4%)	2 (6.5%)	0.045	3 (6.1%)	0 (0%)	0.114
Coronary heart disease	8 (13.6%)	2 (6.5%)	0.484	9 (18.4%)	0 (0%)	0.001
Glomerular filtration rate	85.1 ± 19.7	101.3 ± 13.4	<0.001	89.0 ± 23.1	100.6 ± 24.3	0.019
Autoantibodies						
SCL70	12 (20.3%)	14 (45.2%)	0.014	–	–	–
Centromere	28 (47.5%)	8 (25.8%)	0.046	–	–	–
ssDNA	–	–	–	10 (20.4%)	19 (37.3%)	0.063
dsDNA	–	–	–	11 (22.4%)	27 (52.9%)	0.002
Nucleosomes	–	–	–	7 (14.3%)	15 (29.4%)	0.068
RNP	–	–	–	4 (8.2%)	11 (21.6%)	0.092
Immunosuppressive treatment:						
Duration of CS use (months)	37.2 ± 61.6	13.1 ± 29.8	0.016	25.1 ± 41.8	25.4 ± 57.0	0.978
Cumulative CS dose (g)	8.0 ± 14.8	2.7 ± 7.5	0.030	7.5 ± 16.9	4.8 ± 8.3	0.335
Duration of AZA use (months)	10.0 ± 29.6	7.9 ± 27.7	0.747	21.8 ± 50.5	7.7 ± 25.2	0.086
Cumulative AZA dose (g)	29.4 ± 83.0	23.7 ± 83.0	0.763	83.9 ± 244.6	25.1 ± 79.6	0.115

Values presented as number of patients (percentage) or mean ± standard deviation.

AZA, azathioprine; CS, corticosteroid; dsDNA, double-stranded DNA; SLE, systemic lupus erythematosus; SSc, systemic sclerosis; ssDNA, single-stranded DNA.

^a^Only variables suggested for logistic regression analysis are reported; complete report available in Additional file 2.

**Table 7:** Multivariate logistic regression analysis of predictors of atherosclerotic plaque in systemic sclerosis and systemic lupus erythematosus patients

**Variable**	**Beta coefficient**	**Odds ratio (95% CI)**	***P *** **value**
**Systemic sclerosis (*n*** **= 85)**^**a**^			
Age (per 5 years)	0.904	2.47 (1.61 to 3.78)	<0.001
Nicotine use (per 5 pack-years)	0.554	1.74 (1.13 to 2.68)	0.012
Prednisolone intake (per 5 mg/day over 5 years)	0.940	2.56 (1.09 to 6.04)	0.032
**Systemic lupus erythematosus (*n*** **= 98)**^**b**^			
Age (per 5 years)	0.824	2.28 (1.61 to 3.22)	<0.001
Nicotine use (per 5 pack-years)	0.313	1.37 (1.05 to 1.78)	0.021
ssDNA antibody positive	-1.613	0.20 (0.05 to 0.83)	0.026
Duration of azathioprine use (per 5 years)	0.947	2.58 (0.93 to 7.13)	0.068

CI, confidence interval; ssDNA, single-stranded DNA.

^a^Five observations excluded from model because of missing values. Events per variable: 27/3 = 9. Constant = -10.435. Nagelkerke *R*^2^ = 65.6%. Hosmer–Lemeshow test *P* = 0.147. ^b^Two observations excluded from model because of missing values. Events per variable: 49/4 = 12.25. Constant = -8.507. Nagelkerke *R*^2^ = 65.2%. Hosmer–Lemeshow test *P* = 0.914.

### Comparison of baseline carotid and femoral artery duplexsonography results in SLE patients

#### Comparison of carotid and femoral artery intima-media thickness in SLE patients

As shown above in SSc patients we also found in SLE patients a high correlation of IMT between left and right sides at the level of the CCA (*r* = 0.792; *P* < 0.001) and the CFA (*r* = 0.870; *P* < 0.001). Again the correlation between IMT of the CCA and IMT of the CFA was much weaker (*r* = 0.476; *P* < 0.001). There was no significant difference between mean IMT of the CCA and the CFA (0.70 ± 0.13 mm vs. 0.71 ± 0.15 mm; *P* = 0.215), or between left and right IMT of the CCA (0.70 ± 0.14 mm vs. 0.70 ± 0.13 mm; *P* = 0.812) or left and right IMT of the CFA (0.72 ± 0.15 mm vs. 0.71 ± 0.15 mm; *P* = 0.225).

#### Comparison of carotid and femoral artery definitive atherosclerosis (plaques) in SLE patients

In 49 SLE patients, a total of 89 carotid artery plaques and 69 femoral artery plaques were detected. Forty-seven carotid artery plaques were found in 31 patients on the left side compared with 42 plaques in 33 patients on the right side (31% vs. 33%, *P* = 0.789). The distribution of carotid artery plaques according to the CCA, region of the bulb, ICA and ECA is shown in Figure 1. In 6 to 8% of patients, carotid plaques would have been not detected if carotid ultrasound examination was limited to one side.

Thirty-four femoral artery plaques were found in 25 patients on the left side and 35 plaques in 30 patients on the right side (25% vs. 30%, *P* = 0.301). In 5 to 12% of patients the plaques would have been missed if femoral ultrasound examination was limited to one side. Twenty-four patients had atherosclerotic plaques in only one of the four vascular territories. The overall frequency of carotid and femoral artery plaques was not different (39 vs. 35%, *P* = 0.541), but plaque distribution varied in individual patients: in 14% of patients, plaque was only located at the carotid arteries; in 10% of patients, plaque was observed isolated at femoral arteries. Thus, in one out of 10 patients atherosclerosis would have been not detected if routine ultrasonography was limited to the carotid arteries.

#### Comparison of different pathologic intima-media definitions for the common carotid artery with the presence of atherosclerotic plaque in SLE patients

There was high incongruity in the presence of atherosclerotic plaques and a pathologic thickened intima-media, independent of the criteria used to define pIMT. IMT > 0.9 mm was detected in only 13 out of 49 SLE patients (26.5%) with atherosclerotic plaques. Moreover, according to the European definition as well as the German definition, none of the patients without atherosclerotic plaques had a pathologic intima-media. Using the ARIC definition (taking note of individual difference in IMT according to sex and increasing IMT with age), 5.9% of SLE patients without atherosclerotic plaques had a pIMT. Subgroup analysis revealed that all SLE patients with baseline coronary heart disease, pulmonary hypertension and/or peripheral vascular disease have had atherosclerotic plaques, but in contrast only one out of two or three patients had a thickened intima media (Table 2).

### Association of potential risk factors with intima-media thickness and atherosclerotic plaques in SLE patients

#### Correlation of traditional cardiovascular risk factors and disease-related factors with intima-media thickness in SLE patients

Correlations between common carotid and femoral artery IMT with traditional and nontraditional risk factors for atherosclerosis are shown in Table 4 (and Additional file 1). In multivariate analysis, no variable other than age was independently associated with IMT of the CCA or CFA in SLE patients and multivariate models performed no better than simple linear regression. Thus, instead of a multivariate linear regression model we reported the result of the simple linear regression (Table 5).

#### Association of traditional cardiovascular and disease-related risk factors with the presence of atherosclerotic plaques in SLE patients

Detailed results of univariate analysis are presented in Table 6 (and Additional file 2). Interestingly, in multivariate logistic regression analysis, beside the traditional cardiovascular risk factors of age and cumulative nicotine pack-years, the duration of AZA use also seemed to be independently associated with presence of atherosclerotic plaque. Out of the autoantibodies, only ssDNA but not dsDNA, nucleosomes or RNP antibodies stayed in the final "best" model, and presence of ssDNA autoantibodies was independently associated with the absence of atherosclerotic plaques (Table 7).

### Longitudinal analysis of cardiovascular events during follow-up in SSc and SLE patients

Overall, follow-up data of CVEs were available for 78/90 (87%) SSc patients and 51/100 (51%) SLE patients. During the observational period of in a total 650.1 person-years (mean 60.5 months, median 65.5 months, range 8 to 82 months), 24 CVEs were recorded in 19 out of 129 patients (15%): 13 events in 11/78 (14%) SSc patients (coronary events, *n* = 7; peripheral arterial vascular events, *n* = 3; death related to cardiovascular disease, *n* = 3) and 11 CVEs in 8/51 (16%) SLE patients (coronary events, *n* = 5; cerebrovascular events, *n* = 3; peripheral arterial vascular events, *n* = 3). The incidence of CVEs was 13 per 388.5 person-years (IDR = 3.35 per 100 person-years) in SSc patients and 11 per 261.6 person-years (IDR = 4.2 per 100 person-years) in SLE patients. Six patients (five SSc and one SLE) died due to other causes (one after stem cell transplantation, one severe pneumonia, one lung embolism, one breast cell cancer, one suicide, one unknown cause).

According to pathologic IMT of the CCA, only 7/19 (36.8%) patients with CVEs had pIMT according to the European definition (>0.9 mm), only 4/19 (21.1%) patients would meet the ARIC definition, and merely 2/19 (10.5%) patients the German definition of pathologic IMT.

**Figure 2:**
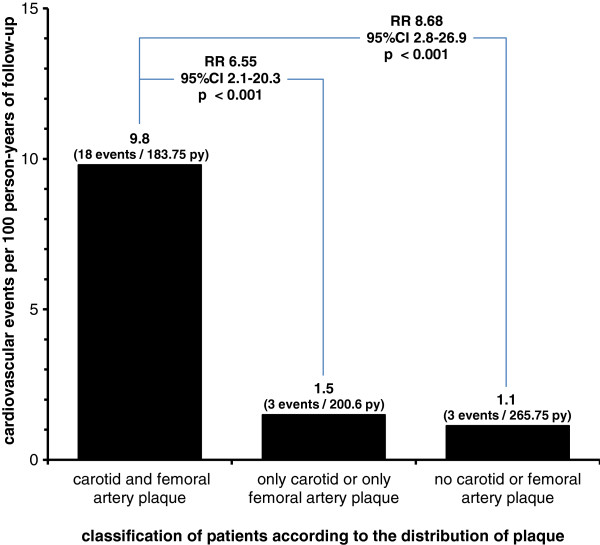
**Incidence and rate ratios of cardiovascular events during follow-up according to the presence of plaque.** Incidence of cardiovascular events per 100 person-years (py) during follow-up available for 129/190 (67.9%) patients (650 person-years, mean 60.5 months, median 65.5 months, range 8 to 82 months) according to the presence of carotid and femoral artery plaque. The rate ratio (RR) was approximately 6.5 for patients with carotid and femoral artery plaque in contrast to patients with only one vascular segment affected and was 8.7 compared with patients without plaque. CI, confidence interval.

According to the presence of plaque, CVEs were distributed as follows: 18 CVEs were observed within 183.75 person-years of follow-up (IDR = 9.8 per 100 person-years) in 15 out of 40 patients (37.5%) that had both carotid and femoral artery plaques; three CVEs per 200.6 person-years (IDR = 1.5 per 100 person-years) occurred in 2/37 (5.4%) patients that had only carotid (1/19, 5.2%) or only femoral artery plaques (1/18, 5.6%); and three CVEs per 265.75 person-years (IDR = 1.1 per 100 person-years) in 2/52 patients (3.8%) that had neither carotid nor femoral artery plaques. The incidence rate of CVEs during follow-up was thus significantly higher in patients with carotid and femoral artery plaque compared with patients with only carotid or only femoral artery plaque (rate ratio = 6.55, 95% CI = 1.9 to 22.2, *P* = 0.003) or compared with patients without atherosclerotic plaque (rate ratio = 8.68, 95% CI = 2.6 to 29.5, *P* < 0.001) (Figure 2). Patients with only carotid plaque or only femoral plaque were not at higher risk compared with patients without plaque (rate ratio = 1.32, 95% CI = 0.3 to 6.6, *P* = 0.731).

**Figure 3:**
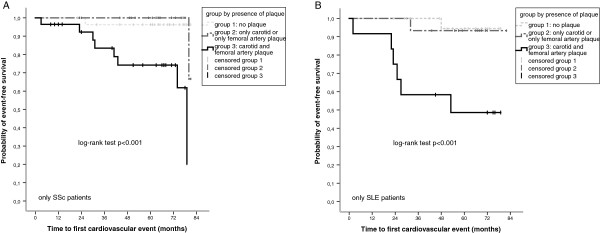
**Kaplan–Meier survival curves for the time to first cardiovascular event according to the presence of plaque.** Kaplan–Meier survival curves for the time to first cardiovascular event during follow-up and results of a log-rank test stratified by **(A)** systemic sclerosis (SSc) and **(B)** systemic lupus erythematosus (SLE) according to the presence of carotid and femoral artery plaques. Group 1, patients without carotid or femoral artery plaque; group 2, patients with only carotid or only femoral artery plaque; group 3, patients with coexistent carotid and femoral artery plaque.

Analysis of time to first CVE revealed that SSc and SLE patients with coexistent carotid and femoral artery plaque are at higher risk (Figure 3A,B). Results from univariate analysis of potential predictors of CVEs during follow-up in SSc and SLE patients are presented in Table 8.

**Table 8:** **Explorative analysis of potential predictors of cardiovascular events during follow-up in systemic sclerosis and systemic lupus erythematosus patients**^**a**^

	**SSc and SLE (*n*** **= 129)**		
**Variable**	**Beta coefficient**	**Unadjusted hazard ratio (95% CI)**	***P *** **value**
SSc versus SLE	-0.173	0.84 (0.33 to 2.10)	0.712
Age (per 5 years)	0.262	1.30 (1.10 to 1.54)	0.003
Male sex	2.833	17.00 (5.99 to 48.22)	<0.001
Carotid and femoral artery plaque			
Group 3 (CP and FP) versus group 1 (no plaque)	2.595	13.40 (2.99 to 59.95)	<0.001
Group 3 (CP and FP) versus group 2 (CP or FP)	2.672	14.47 (2.51 to 83.55)	0.003
Group 2 (CP or FP) versus group 1 (no plaque)	-0.077	0.93 (0.12 to 7.39)	0.942
Arterial hypertension	1.678	5.35 (1.91 to 15.02)	<0.001
Nicotine use (per 5 pack-years)	0.143	1.15 (1.04 to 1.28)	0.005
Coronary heart disease	2.592	13.36 (4.67 to 38.20)	<0.001
Age at diagnosis (per 5 years)	0.075	1.20 (1.04 to 1.39)	0.014
C-reactive protein (per mg/dl)	0.220	1.25 (0.98 to 1.59)	0.079

CI, confidence interval; CP, carotid artery plaque; FP, femoral artery plaque; SLE, systemic lupus erythematosus; SSc, systemic sclerosis.

^a^Only variables suggested for multivariate Cox regression analysis are reported; complete report available in Additional file 3.

In multivariate Cox regression analysis (model 1), the coexistent presence of carotid and femoral artery plaque was predictive for CVEs during follow-up compared with patients without plaque (adjusted HR = 6.13, 95% CI = 1.27 to 29.53, *P* = 0.024) whereas it was not for patients with only one vascular segment affected compared with patients without plaque (adjusted HR = 1.10, 95% CI = 0.13 to 9.08, *P* = 0.932). If patients with coexistent carotid and femoral artery plaque were compared with patients with only one vascular segment affected, the adjusted HR was 5.59 (95% CI = 0.86 to 36.16, *P* = 0.071). After dichotomization of the variable to improve the Cox regression analysis (model 2), the adjusted HR for CVEs in patients with coexistent carotid and femoral plaques was 5.92 (95% CI = 1.55 to 22.67, *P* = 0.009) compared with other patients (with only carotid plaque, only femoral plaque or without plaque). Additionally, baseline coronary heart disease (adjusted HR = 10.19, 95% CI = 3.04 to 34.17, *P* < 0.001) and male sex (adjusted HR = 8.78, 95% CI = 2.73 to 28.19, *P* < 0.001) were independently associated with incident CVEs whereas arterial hypertension, age, age at diagnosis and nicotine pack-years were associated only in univariate analysis but not multivariate analysis (Table 9).

**Table 9:** Multivariate Cox regression analysis of predictors of incident cardiovascular events during follow-up in systemic sclerosis and systemic lupus erythematosus patients

**Variable**	**Beta coefficient**	**Adjusted hazard ratio (95% CI)**	***P *** **value**
**Cox model 1 – SSc and SLE (*n*** **= 129)**^**a**^			
Coronary heart disease	2.326	10.24 (3.04 to 34.55)	<0.001
Male sex	2.172	8.78 (2.73 to 28.19)	<0.001
Carotid and femoral artery plaque			
Group 3 (CP and FP) versus group 1 (no plaque)	1.812	6.13 (1.27 to 29.53)	0.024
Group 2 (CP or FP) versus group 1 (no plaque)	0.092	1.10 (0.13 to 9.08)	0.932
**Cox model 2 – SSc and SLE (*n*** **= 129)**^**b**^			
Coronary heart disease	2.322	10.19 (3.04 to 34.17)	<0.001
Male sex	2.172	8.78 (2.73 to 28.19)	<0.001
Carotid and femoral artery plaque			
Group 3 (CP and FP) versus all other (CP or FP or no plaque)	1.778	5.92 (1.55 to 22.67)	0.009

Group 1, patients without carotid or femoral artery plaque; group 2, patients with only carotid or only femoral artery plaque; group 3, patients with coexistent carotid and femoral artery plaque. CI, confidence interval; CP, carotid artery plaque; FP, femoral artery plaque; SLE, systemic lupus erythematosus; SSC, systemic sclerosis. ^a^Zero observations excluded from model because of missing values. Events per variable: 19/4 = 4.75. ^b^Zero observations excluded from model because of missing values. Events per variable: 19/3 = 6.33.

## Discussion

### Intima-media thickness as surrogate marker of atherosclerosis in the general population

Measurement of intima-media thickening in the CCA is a widespread accepted surrogate marker for subclinical atherosclerosis [32,33], although the clinical importance for the individual patient was recently challenged in the general population [34,35]. Although the assessment of both IMT and atherosclerotic plaques is recommended for epidemiological trials studying cardiovascular disease, it is important to keep in mind that IMT not only represents subclinical atherosclerosis but is also influenced by nonatherosclerotic remodeling [27]. Presence of plaque is of overriding importance in reflecting cardiovascular risk [36,37].

### What is the rationale to determine a fixed definition of pathological increased intima-media thickness?

Although the term pathologic IMT is widely used, it is not well defined. According to the guidelines of the European Society of Cardiology, pIMT is defined as a focal IMT >0.9 mm, regardless of gender [24]. Different cutoff values were reported in several studies, ranging between IMT ≥1.0 mm and ≥1.18 mm for prediction of CVEs [38-40]. However, a fixed definition does not take regard of an increase in IMT with older age and differences between men and women, which is well documented in large studies [26]. For the German population only one small study reports reference values with respect to age and gender for pIMT: a cIMT > 1.0 mm in men 40 to 70 years old, cIMT > 0.85 mm in women 40 to 54 years old and cIMT > 1.0 mm in women 55 to 70 years old were defined as pIMT [25].

In SLE patients, Thompson and colleagues evaluated a mean IMT progression rate of 0.011 mm/year, which was comparable with the IMT progression rate in controls [4]. These rates also seem comparable with the rate of mean IMT progression in healthy controls evaluated in the large ARIC study of approximately 0.01 mm/year [26]. In our cohort, with respect to the results of baseline linear regression analysis, carotid IMT increased slightly less with 0.007 mm and 0.006 mm per age year in SSc and SLE patients, respectively. For that reason, in addition to the fix definition of pIMT given by the European Society of Cardiology [24], we also used an age-adjusted and sex-adjusted definition of pIMT for the German population [25], and calculated an "ARIC definition" for pIMT, defined by the 90th percentile of a large cohort from the ARIC study [26].

### Is carotid intima-media thickness measurement a useful parameter for atherosclerosis assessment in SSc and SLE patients?

Previous studies in SSc and SLE patients showed a marked heterogeneity in IMT results, raising the question of whether IMT measurement is generally useful in these disease entities. Two recent meta-analyses reported a moderate statistically significant increased IMT in SSc and SLE patients compared with healthy controls [1,2]. However, the clinical relevance of these findings is questionable because the "thickened" IMT of SSc and SLE patients seems to be within the range of normal values, and the mean IMT of controls was surprisingly low in some studies (and none of these studies analyzed the predictive value for CVEs). Moreover, most of the included studies in these meta-analyses primarily searched for a difference in IMT in the CCA, but atherosclerotic plaques at the bulb, ICA and ECA were not evaluated. Additionally, it is unclear whether other studies were not published because they also primarily focused on cIMT without looking for plaques and found no difference in cIMT (publication bias).

In our study we were able to show in both SSc and SLE patients that atherosclerotic plaques frequently occur without a thickened intima-media, regardless of how the definition of pIMT was applied. Our observed frequency of atherosclerotic plaques is consistent with studies of larger patient cohorts in SSc [10,12,15] and SLE [3-5].

The currently largest case–control study in 111 SSc patients by Nordin and colleagues reported a 48% frequency of plaques in patients compared with 41% in controls [10], but the prevalence of atherosclerotic plaques in SSc might have been underestimated when ultrasound examination did not include the ICA and ECA and by application of an unusual definition of plaque as IMT >1 mm and 100% increase of IMT compared with the adjacent wall. In contrast, Lukjanowicz and colleagues reported an increased frequency of atherosclerotic plaques in 25 of 62 SSc patients (40.3%) compared with no plaques in 30 healthy controls, also without any signs of intima-media thickening in SSc patients, which supports our observations (results published as abstract, details of ultrasound examination are unknown) [11,12].

In SLE patients, Roman and colleagues (*n* = 197) [3], Thompson and colleagues (*n* = 217) [4] and Anania and colleagues (*n* = 114) [5] reported a frequency of 37 to 43% carotid artery plaques compared with 15 to 30% in healthy controls, and found no difference [4,5] or even a lower cIMT [3] in SLE patients. In large studies without comparison with healthy controls, Manzi and colleagues (*n* = 175) [41] and Kao and colleagues (*n* = 392) [20] have observed carotid plaques in 30 to 40% of SLE patients.

Carotid atherosclerosis is thus frequent and may have been previously underestimated in both connective tissue diseases, when only cIMT was measured. An extension of carotid ultrasound examination distal to the CCA segment for plaque assessment is highly recommended, because the majority of plaques are localized distant to the CCA (only ~5% of plaques) at the region of the bulb (~40%), ICA (~45%) and ECA (~10%). Additionally, the use of a common definition of atherosclerotic plaque would be desirable to achieve comparable results between further studies [27].

### Is carotid intima-media thickness measurement a useful parameter for prediction of cardiovascular events in SSc and SLE patients?

In general, the discrepancy between manifest atherosclerotic plaques and the absence of intima-media thickening raises the question of whether IMT measurement is useful in clinical routine care of SSc and SLE patients. With respect to clinically relevant atherosclerosis (that is, predictive of CVEs), IMT measurement was not as useful as detection of plaques for predicting CVEs in our cohort. Even if patients with incident CVEs have a higher baseline cIMT, as observed in a one large cohort of SLE patients (0.80 vs. 0.64 mm) [20], they would not have been identified as at risk of CVEs in routine clinical care if this increase does not fulfill the definition of pathologic IMT. Moreover, in accordance with our results, this large cohort study by Kao and colleagues in SLE patients revealed that assessment of plaques is more important than measurement of IMT for risk prediction of incident CVEs (HR 4.26 vs. 1.35, respectively). In SSc patients, large cohort studies of CVEs including atherosclerotic plaque assessment are lacking. Our data suggest that in SSc patients the detection of atherosclerotic plaques but not IMT is of clinical relevance in predicting CVEs as well.

### Is additive femoral artery sonography useful to evaluate atherosclerosis and to identify SSc and SLE patients at risk of cardiovascular events?

Our results of plaque localization, representing differences between left and right sides as well as carotid and femoral artery plaque presence, underline the results of Belcaro and colleagues [16], who showed the necessity of bilateral carotid and femoral artery examination to estimate the presence of atherosclerosis accurately (in accordance with carotid arteries, the evaluation of atherosclerotic plaques seems to be more useful than the evaluation of IMT in femoral arteries). Furthermore, in concordance with the results of Belcaro and colleagues [16], the combined assessment of carotid and femoral artery plaques predicted significantly more CVEs during follow-up than the measurement of only one vascular segment. With respect to confounder-adjusted hazards, patients with coexistent carotid and femoral artery plaque had an approximate HR of 6 in contrast to patients with only carotid or only femoral artery plaque as well as in contrast to patients without plaque. In comparison, Kao and colleagues observed a HR of 4.3 for carotid plaque predictive of "hard" CVEs in SLE patients [20] but did not assess the femoral arteries. In our cohort of SSc and SLE patients, if we did not account for coexistent femoral artery atherosclerosis, we would have estimated a confounder-adjusted HR of 3.5 for carotid plaque predictive of CVEs (data shown in Additional file 3). The risk of CVEs by presence of carotid artery plaque would thus have been overestimated in patients without plaque and underestimated in patients with unknown coexistence of femoral artery plaque. Taken together, additional screening of the femoral artery is very useful to identify SSc and SLE patients at high risk.

### Association of atherosclerosis with traditional and nontraditional cardiovascular risk factors, immunosuppressive medications and SSc and SLE specific variables

In SLE, previous studies have reported several traditional cardiovascular risk factors such as age, male sex, arterial hypertension, obesity, dyslipidemia and family history, but also disease-related factors such as disease duration, disease activity, damage score, renal involvement and prednisolone use to be associated with atherosclerosis [3,4,42-53]. There is still a controversial debate as to whether accelerated atherosclerosis is or is not a feature of SSc [8,9]. However, in studies indicating a higher prevalence of atherosclerosis in SSc, an association with age, arterial hypertension, triglyceride levels, smoking, disease duration and cumulative CS dosage was observed [10,54,55]. In our cohort in univariate analysis, age, male gender, postmenopausal status in women, arterial hypertension, dyslipidemia and nicotine pack-years were associated with atherosclerotic plaques. Moreover, the presence of plaque was associated with a higher age at diagnosis, but not with disease duration in both patient groups. There was no association with illness-specific activity and damage scores such as the Systemic Lupus Erythematosus Disease Activity Index and the Systemic Lupus International Collaborating Clinics damage index in SLE, or the modified Rodnan Skin Score in SSc patients. Notably, age was the strongest factor associated with CCA and CFA IMT. Furthermore, postmenopausal status correlated with CCA IMT, and nicotine pack-years with CFA IMT, whereas other cardiovascular risk or disease-related factors did not. In multiple logistic regression analysis, age and nicotine pack-years were independently associated with atherosclerotic plaque occurrence over all patients. Additionally, in SSc patients the cumulative prednisolone dose was associated with atherosclerotic plaque, supporting the idea that prednisone use may be a deteriorating factor for atherosclerosis in SSc [55]. In SLE patients, corticosteroids have shown to increase cardiovascular risk factors such as arterial hypertension and body weight, whereas HCQ leads to a decrease of serum cholesterol levels [56]. Moreover, CYP, HCQ and CS use seem to be associated with a lower frequency of atherosclerotic plaque in SLE patients [49]. However, in our smaller cohort we found no significant differences in CYP, HCQ or CS medication between SLE patients with or without atherosclerotic plaque.

Interestingly, in multivariate analysis AZA use seemed to be associated with atherosclerotic plaques in SLE patients. AZA was recently found to be associated with an increased cIMT in pediatric SLE patients [47] and with vascular events in adult SLE patients [57]. Because of the limited number of SLE patients available for follow-up analysis in our cohort we were not able to analyze AZA as contributing risk factor for CVEs.

### Association of autoantibodies with atherosclerosis in SSc and SLE

High titers of antinuclear antibodies were found to be associated with coronary atherosclerosis [58]. In our cohort, current or former ANA were not associated with IMT, and neither were with the presence of atherosclerotic plaque. Antiphospholipid antibodies are supposed to activate endothelial cells and are hence suspected to accelerate the atherosclerosis process [59], and different other autoantibodies were reported to correlate with cardiovascular disease in SLE. Roman and colleagues found anti-Sm, anti-RNP and anticardiolipin antibodies significantly more often in SLE patients without compared with those with carotid artery plaque [49]. Similarly, other studies reported that Sm antibodies may have cardioprotective effects and were less often observed in SLE patients with coronary atherosclerosis or carotid artery plaque [52,60]. In our cohort, we found no difference in atherosclerotic plaque frequency between SLE patients with or without anti-Sm or anticardiolipin antibodies. Recently, dsDNA antibodies were suggested to contribute to noncalcified coronary plaque [44]. In contrast, in our cohort dsDNA antibodies were found less frequently in patients with atherosclerotic plaques in univariate analysis, but were not confirmed in multivariate analysis (which can be explained by the fact that dsDNA antibodies occurred more often in older SLE patients in our cohort). Surprisingly, in multivariate analysis absence of the less disease-specific ssDNA antibodies did qualify as a statistically significant independent predictor for atherosclerotic plaque in SLE patients. We found no confounder that can explain this unexpected finding; this observation may be verified in further studies.

Atherosclerosis was found more often in anti-centromere antibody-positive SSc patients [10], and coronary artery calcification less often in anti-SCL70 antibody-positive SSc patients [61]. Coronary flow reserve was reported to be lower in diffuse cutaneous SSc patients [62], perhaps due to microvascular involvement rather than atherosclerosis [8]. In SSc patients of our cohort, atherosclerosis was also found more often in the limited than diffuse cutaneous subtype, hence centromere antibody was found more often and SCL70 antibody less often in patients with atherosclerotic plaque. However, these findings in our cohort were due to the older age of patients with centromere antibodies compared with patients with Scl-70 antibodies; after adjustment for age there was no statistically significant difference (data not shown).

### Limitations of the study

Whether the number of patients with plaque from the SSc and SLE patients was high enough to build two separate logistic regression models with a suitable number of events per variable, it was not for Cox regression and the analysis of both diseases was combined to ensure at least five patients with CVEs per variable [63,64]. We are confident that this combined analysis was feasible because we did not observe a significant difference in the frequency or distribution of atherosclerotic plaque nor in the incidence of CVEs between SSc and SLE patients. However, we were not able to differentiate potential risk factors for CVEs between SSc and SLE patients.

The higher follow-up rate of SSc patients (87%) than SLE patients (51%) might be explained by the inclusion of SSc patients in the German network for SSc. Because the loss of follow-up in SLE patients (49%) could have biased our findings, we analyzed whether risk factors for CVEs were more or less prevalent in SLE patients without follow-up: SLE patients available for follow-up analysis had at baseline a slightly shorter disease duration (mean 5 years vs. 6 years), no difference in SLICC damage index score (median 2 vs. 2) and a slightly higher SLEDAI (median 12 vs. 8) compared with patients without follow-up. Baseline coronary heart disease was prevalent in 3/51 (5.9%) SLE patients available versus 6/49 (12.2%) SLE patients nonavailable for follow-up, male sex in 4/51 (7.8%) versus 9/49 (18.4%) and coexistence of carotid and femoral artery plaques in 12/51 (23.5%) versus 13/49 (26.5%) patients, respectively. SLE patients without follow-up were thus most probably at a slightly higher risk for CVEs and we might have underestimated the true incidence of CVEs in SLE patients. On the other hand, the incidence of CVEs in our SLE cohort was higher compared with previous larger cohort studies of SLE patients [18-20]. This difference might be based on unequal study populations, due to a varying definition of CVEs, and patients without follow-up may have been more likely than those without development of CVEs or who died due to other causes (in this scenario we might have overestimated the "true" incidence of CVEs in SLE patients).

However, besides these limitations our study primarily focused on the value of duplexsonography evaluation and demonstrated the uselessness of IMT measurement in contrast to plaque assessment and highlighted the value of additive femoral artery screening for assessment of atherosclerosis and to identify SSc and SLE patients at high risk of incident CVEs. Larger prospective studies are required to estimate the "true" incidence of CVEs and to evaluate further potential risk factors for CVEs, particularly in SSc patients.

## Conclusions

In summary, our study demonstrates a high frequency of atherosclerotic plaques in the carotid arteries as well as in the femoral arteries in the absence of a pIMT in both SSc and SLE patients. IMT measurement appears to be less useful than searching for atherosclerotic plaques in SSc and SLE patients in routine cardiovascular assessment. Sonography of both carotid and femoral arteries to assess atherosclerotic plaques provides a more accurate evaluation of clinically relevant atherosclerosis than only carotid artery screening in order to reduce the overall morbidity and mortality in affected patients.

## Abbreviations

ARIC: Atherosclerosis Risk in Communities; AZA: azathioprine; CCA: common carotid artery; CFA: common femoral artery; CI: confidence interval; cIMT: carotid intima-media thickness; CS: corticosteroid; CVE: cardiovascular event; CYP: cyclophosphamide; dsDNA: double-stranded DNA; ECA: external carotid artery; HCQ: hydroxychloroquine; HR: hazard ratio; ICA: internal carotid artery; IDR: incidence density rate; IMT: intima-media thickness; pIMT: pathologic intima-media thickening; SLE: systemic lupus erythematosus; SSc: systemic sclerosis; ssDNA: single-stranded DNA.

## Competing interests

The authors declare that they have no competing interests. This research received no specific grant from any funding agency in the public, commercial, or not-for-profit sectors.

## Authors’ contributions

MF and SMW conceived of the study, JS participated in the design of the study. All authors were involved in the recruitment and the clinical examination of patients. AK and SMW assessed the skin thickening for the modified Rodnan Skin Score. JS and SMW performed the ultrasound examinations. MF evaluated the stored anonymous images without knowledge of patient data and characteristics. AK and MF collected the data of follow-up for CVEs. MF established the database and performed the statistical analysis, the review of literature and drafted the manuscript. SMW and JS helped to draft the manuscript. All authors read and approved the final manuscript.

## Supplementary Material

Additional file 1 **Is a table presenting correlation of carotid and femoral artery IMT with traditional and nontraditional risk factors for atherosclerosis.** A detailed report of correlations between mean common carotid IMT and mean femoral artery IMT with traditional and nontraditional risk factors for atherosclerosis in SSc and SLE patients. Click here for file

Additional file 2 **Is a table presenting explorative univariate analysis of potential risk factors between patients with or without atherosclerotic plaques.** A detailed report of explorative univariate analysis of the association of traditional cardiovascular and disease-related risk factors with presence of atherosclerotic plaques in SSc and SLE patients. Click here for file

Additional file 3 **Is tables presenting explorative analysis and additional multivariate Cox regression analysis of predictors of incident CVEs during follow-up in SSc and SLE patients.** The first table shows a detailed report of explorative analysis of predictors of CVEs during follow-up in SSc and SLE patients. The second table shows additional multivariate Cox regression analysis of predictors of incident CVEs during follow-up in SSc and SLE patients including only carotid but not femoral artery plaques (if only carotid artery duplexsonography was performed and status of femoral artery plaque was unknown). Click here for file
