# Supplementary material for: Atherosclerotic plaques occur in absence of intima-media thickening in both systemic sclerosis and systemic lupus erythematosus: a duplexsonography study of carotid and femoral arteries and follow-up for cardiovascular events
Source: Arthritis Res Ther. 2014 Feb 19;16(1):R54. doi: 10.1186/ar4489 (PMC3978872; doi:10.1186/ar4489)
Supplement: Additional file 1 — Is a table presenting correlation of carotid and femoral artery IMT with traditional and nontraditional risk factors for atherosclerosis. A detailed report of correlations between mean common carotid IMT and mean femoral artery IMT with traditional and nontraditional risk factors for atherosclerosis in SSc and SLE patients. [file ar4489-S1.pdf]

Additional file 1: Correlation of carotid and femoral artery IMT with traditional and non-traditional risk factors for atherosclerosis

Variable	All patients (n=190)				SSc (n=90)				SLE (n=100)			
	mIMT CCA		mIMT CFA		mIMT CCA		mIMT CFA		mIMT CCA		mIMT CFA	
	correlation	p-value	correlation	p-value	correlation	p-value	correlation	p-value	correlation	p-value	correlation	p-value
Age, years	r=0.717	<0.001	r=0.581	<0.001	r=0.682	<0.001	r=0.565	<0.001	r=0.726	<0.001	r=0.560	<0.001
Age at diagnosis, years	r=0.677	<0.001	r=0.538	<0.001	r=0.652	<0.001	r=0.586	<0.001	r=0.670	<0.001	r=0.437	<0.001
Sisease duration, months	r=0.047	0.524	r=0.046	0.443	r=-0.030	0.782	r=-0.109	0.308	r=0.094	0.357	r=0.217	0.032
Male sex	rφ=-0.259	<0.001	rφ=0.272	<0.001	rφ=0.261	0.013	rφ=0.341	0.001	rφ=0.268	0.007	rφ=0.208	0.039
Postmenopausal status	r=0.487	<0.001	r=0.389	<0.001	r=0.546	0.001	r=0.375	0.001	r=0.400	<0.001	r=0.373	<0.001
Body-mass index	r=0.170	0.023	r=0.220	0.003	r=0.128	0.241	r=0.197	0.070	r=0.258	0.012	r=0.294	0.004
Systolic blood pressure, mmHg	r=0.314	<0.001	r=0.251	0.001	r=0.303	0.007	r=0.332	0.003	r=0.346	0.001	r=0.171	0.124
Diastolic blood pressure, mmHg	r=0.121	0.121	r=0.064	0.424	r=0.067	0.564	r=0.128	0.266	r=0.228	0.029	r=0.024	0.833
Triglyceride, mg/dl	r=-0.020	0.784	r=0.087	0.236	r=-0.137	0.199	r=-0.040	0.706	r=0.099	0.333	r=0.235	0.021
Total Cholesterol, mg/dl	r=0.031	0.672	r=-0.039	0.596	r=0.035	0.743	r=-0.052	0.628	r=0.027	0.790	r=-0.030	0.767
Low density lipoprotein, mg/dl	r=0.033	0.650	r=0.033	0.658	r=.0-008	0.940	r=0.057	0.593	r=0.065	0.523	r=0.004	0.966
High density lipoprotein, mg/dl	r=-0.012	0.876	r=-0.033	0.654	r=0.049	0.652	r=0.112	0.300	r=-0.072	0.486	r=-0.195	0.057
Glomerular filtration rate	r=-0.313	<0.001	r=-0.379	<0.001	r=-0.316	0.003	r=-0.489	<0.001	r=-0.296	0.003	r=-0.277	0.006
Nicotine pack years	r=0.049	0.509	r=0.154	0.036	r=0.022	0.839	r=0.320	0.002	r=0.082	0.424	r=-0.058	0.570
Coronary heart disease	rφ=0.211	0.004	rφ=0.281	<0.001	rφ=0.160	0.133	rφ=0.379	<0.001	rφ=0.265	0.008	rφ=0.160	0.115
Peripheral arterial vascular disease	rφ=0.043	0.560	rφ=0.112	0.124	rφ=-0.009	0.930	rφ=.117	0.270	rφ=.081	0.426	rφ=0.060	0.557
Pulmonary arterial hypertension	rφ=0.188	0.010	rφ=0.093	0.201	rφ=0.140	0.189	rφ=0.076	0.476.	rφ=0.172	0.088	rφ=0.0.28	0.783
Diabetes mellitus	rφ=0.044	0.552	rφ=0.197	0.007	rφ=0.023	0.832	rφ=0.345	0.001	rφ=0.080	0.434	rφ=.082	0.417
SLEDAI	-	-	-	-	-	-	-	-	r=-0.166	0.115	r=-0.057	0.591
SLICC	-	-	-	-	-	-	-	-	r=0.208	0.049	r=0.117	0.271
mRSS	-		-	-	r=0.225	0.036	r=-0.021	0.843	-	-	-	-

C3, mg/dl	r=0.113	0.152	r=0.074	0.351	r=-0.032	0.787	r=0.023	0.847	r=0.159	0.142	r=0.065	0.547
C4, mg/dl	r=0.129	0.118	r=0.055	0.512	r=0.082	0.499	r=0.168	0.162	r=0.158	0.173	r=-0.001	0.993
CRP, mg/dl	r=0.097	0.233	r=0.056	0.493	r=0.019	0.873	r=-0.096	0.427	r=0.181	0.101	r=0.174	0.116
5-yr daily dose of prednisone, mg	r=-0.103	0.172	r=-0.045	0.553	r=-0.074	0.498	r=-0.041	0.709	r=-0.150	0.154	r=-0.065	0.539
Duration of CS use, months	r=0.039	0.600	r=0.034	0.647	r=0.028	0.794	r=0.097	0.370	r=0.035	0.733	r=-0.058	0.576
Cumulative CS dose, g	r=-0.073	0.334	r=0.005	0.945	r=-0.042	0.699	r=-0.044	0.688	r=-0.110	0.296	r=0.060	0.568
Duration of HCQ use, months	r=0.023	0.754	r=-0.026	0.725	r=0.045	0.672	r=0.030	0.779	r=0.107	0.295	r=0.029	0.778
Cumulative HCQ dose, g	r=0.037	0.611	r=-0.060	0.414	r=0.045	0.671	r=0.022	0.839	r=0.124	0.225	r=-0.031	0.761
Duration of AZA use ,months	r=-0.017	0.817	r=0.037	0.611	r=-0.040	0.709	r=-0.029	0.788	r=0.029	0.778	r=0.095	0.354
Cumulative AZA dose, g	r=0.009	0.899	r=0.015	0.843	r=0.015	0.891	r=-0.028	0.795	r=0.041	0.688	r=0.072	0.481
Duration of CYP use, months	r=-0.053	0.466	r=0.003	0.972	r=-0.121	0.256	r=-0.107	0.315	r=-0.028	0.787	r=0.057	0.578
Cumulative CYP dose, g	r=-0.071	0.332	r=-0.019	0.795	r=-0.131	0.217	r=-0.097	0.364	r=-0.061	0.553	r=0.013	0.903
Antiphospholipid antibodies	rφ =-0.054	0.458	rφ =-0.121	0.097	rφ =-0.118	0.270	rφ =-0.168	0.113	rφ =0.033	0.749	rφ =-0.053	0.601
ANA, current or former	rφ =-0.038	0.599	rφ =-0.035	0.632	rφ =-0.035	0.740	rφ =-0.041	0.704	rφ=-0.066	0.517	rφ=-0055	0.588
SSA-ab	rφ=-0.179	0.014	rφ=-0.202	0.005	rφ=-0.149	0.162	rφ=-0.178	0.094	rφ=-0.125	0.219	rφ=-0.154	0.127
SSB-ab	rφ=-0.065	0.372	rφ=-0.155	0.033	rφ=-0.054	0.611	rφ=-0.094	0.377	rφ=-0.008	0.938	rφ=-0.151	0.136
ssDNA-ab	rφ=-0.091	0.212	rφ=-0.080	0.276	-	-	-		rφ=0.014	0.893	rφ=-0.019	0.850
dsDNA-ab	rφ=-0.210	0.004	rφ=-0.180	0.013	-	-	-	-	rφ=-0.188	0.063	rφ=-0.197	0.051
SM-ab	rφ=-0.102	0.650	rφ=-0.042	0.564	-	-	-	-	rφ=-0.057	0.573	rφ=-0.023	0.824
Nukleosomes-ab	rφ=-0.215	0.003	rφ=-0.141	0.052	-	-	-	-	rφ=-0.231	0.021	rφ=-0.123	0.224
RNP-ab	rφ=-0.094	0.200	rφ=-0.091	0.212	-	-	-	-	rφ=-0.058	0.569	rφ=-0.044	0.668
Centromere-ab	rφ=0.154	0.034	rφ=0.219	0.002	rφ=0.165	0.119	rφ=0.255	0.015	-	-	-	-
SCL70-ab	rφ=-0.010	0.888	rφ=-0.071	0.335	rφ=-0.117	0.270	rφ=-0.209	0.048	-	-	-	-

IMT = intima-media thickness, CCA = common carotid artery, CFA = common femoral artery, mRSS = modified Rodnan Skin Score, SLEDAI = Systemic Lupus Erythematosus Disease Activity Index, SLICC = Systemic Lupus International Collaborating Clinics damage index, CS = corticosteroid, HCQ = hydroxychloroquine, AZA = azathioprine, CYP = cyclophosphamid
