# Supplementary material for: Atherosclerotic plaques occur in absence of intima-media thickening in both systemic sclerosis and systemic lupus erythematosus: a duplexsonography study of carotid and femoral arteries and follow-up for cardiovascular events
Source: Arthritis Res Ther. 2014 Feb 19;16(1):R54. doi: 10.1186/ar4489 (PMC3978872; doi:10.1186/ar4489)
Supplement: Additional file 2 — Is a table presenting explorative univariate analysis of potential risk factors between patients with or without atherosclerotic plaques. A detailed report of explorative univariate analysis of the association of traditional cardiovascular and disease-related risk factors with presence of atherosclerotic plaques in SSc and SLE patients. [file ar4489-S2.pdf]

Additional file 2: Explorative univariate analysis of potential risk factors between patients with or without atherosclerotic plaques

Characteristic	SLE and SSc (n=190)			SSc (n=90)			SLE (n=100)		
	with Plaque (n=108)	without Plaque (n=82)	p-value	with Plaque (n=59)	without Plaque (n=31)	p-value	with Plaque (n=49)	without Plaque (n=51)	p-value
Age, years	61.0 ± 11.8	41.6 ± 12.6	<0.001	63.9±12.0	45.9±10.9	<0.001	57.7 ± 10.6	39.0 ± 13.0	<0.001
Male sex	21 (19,4%)	4 (4,9%)	0.004	10 (16.9%)	2 (6.5%)	0.206	11 (22.4%)	2 (3.9%)	0.007
Mean IMT CCA	0.79 ± 0.14	0.64 ± 0.08	<0.001	0.81±0.15	0.66±0.09	<0.001	0.77 ± 0.12	0.62 ±0.08	<0.001
Mean IMT CFA	0.82 ± 0.16	0.65 ± 0.11	<0.001	0.85±0.17	0.65±0.12	<0.001	0.78 ±0.15	0.65 ± 0.11	<0.001
Postmenopausal status, women only	75/87 (86.2%)	28/78 (35.9%)	<0.001	44/49 (89.8%)	12/29 (41.4%)	<0.001	31/38 (81.6%)	16/49 (32.7%)	<0.001
Body-mass index	25.1 ± 4.3	24.5 ± 4.6	0.377	24.9±4.2	23.6±3.7	0.164	25.4 ± 4.4	25.1 ± 5.0	0.760
Body-mass index > 30 (adipositas)	11 (10.2%)	9 (11.0%)	0.860	5 (8.5)	1 (3.2%)	0.660	6 (12.2%)	8 (15.7%)	0.620
Hypertension	77 (71.3%)	24 (29.3%)	<0.001	38 (64.4%)	7 (22.6%)	<0.001	39 (79.6%)	17 (33.3%)	<0.001
- systolic blood pressure, mmHg	137.2 ± 21.6	120.1 ± 19.3	<0.001	136.0±22.9	116.3±17.9	<0.001	138.6±20.1	122.3 ±20.0	<0.001
- diastolic blood pressure, mmHg	78.8 ± 10.5	73.8 ± 11.2	0.005	77.9±11.7	71.5±9.7	0.024	79.9 ± 8.6	75.2 ± 11.9	0.045
- median number of antihypertensives (range)	1 (6)	0 (3)	<0.001	1 (4)	0 (2)	0.001	1 (6)	0 (3)	<0.001
Smoking, current or former	56 (51.9%)	40 (48.8%)	0.675	29 (49.2%)	14 (45.2%)	0.719	27 (55.1%)	26 (51%)	0.860
- nicotine pack years	14.3 ± 21.6	5.4 ± 8.5	<0.001	13.9±22.5	4.1±7.8	0.003	14.7 ±20.6	6.2 ± 8.9	0.009
Diabetes	8 (7.4%)	2 (2.2%)	0.192	4 (6.8%)	0 (0%)	0.294	4 (8.2%)	2 (3.9%)	0.432
Dyslipidemia	54 (50%)	22 (26.5%)	<0.001	30 (50.8%)	7 (22.6%)	0.010	24 (49%)	15 (29.4%)	0.045
Triglyceride, mg/dl	137.8 ± 81,5	127.5 ± 71.5	0.367	142.4±92.8	133.1±68.6	0.625	132.4±66.4	123,9±73.8	0.551
Total Cholesterol, mg/dl	217.6 ± 52.0	211.8 ± 59.3	0.476	216.1±55.5	217.4±38.8	0.909	219.5±47.9	208.4±69.2	0.357
- Low density lipoprotein, mg/dl	127.0 ± 41.3	120.1 ± 45.7	0.281	125.2±42.9	126.0±31.7	0.934	129.0±39.6	116.4±52.6	0.182
- High density lipoprotein, mg/dl	64.2 ± 17.7	63.9 ± 19.0	0.927	63.2±18.2	64.8±19.9	0.701	65.4±17.3	63.4±18.6	0.585
Family history cardiovascular events	15 (13.9%)	13 (15.9%)	0.705	7 (11.9%)	5 (16.1%)	0.572	8 (16.3%)	8 (15.7%)	0.930
Age at diagnosis, years	53.7 ± 14.5	35.4 ± 11.4	<0.001	56.0 ± 14.3	38.7 ± 10.9	<0.001	50.8 ± 14.4	33.4 ± 11.4	<0.001

Disease duration since diagnosis, months	83.3 ± 93.7	68.6 ± 94.2	0.287	87.9 ± 95.2	79.4 ± 98.7	0.689	77.6 ± 92.5	62.1 ± 91.7	0.403
Raynaud phenomenon	76 (70.4%)	47 (57.3%)	0.062	55 (93.2%)	29 (93.5%)	1.000	21 (42.9%)	18 (35.3%)	0.438
Pulmonary hypertension	18 (16.7%)	2 (2.4%)	<0.001	15 (25.4%)	2 (6.5%)	0.045	3 (6.1%)	0 (0%)	0.114
Coronary heart disease	17 (15.7%)	2 (2.4%)	0.003	8 (13.6%)	2 (6.5%)	0.484	9 (18.4%)	0 (0%)	0.001
Peripheral arterial vascular disease	6 (5.6%)	1 (1.2%)	0.143	4 (6.8%)	1 (3.2%)	0.656	2 (4.1%)	0 (0%)	0.238
Glomerular filtration rate	86.8 ± 21.2	100.8 ± 20.8	<0.001	85.1 ± 19.7	101.3 ± 13.4	<0.001	89.0 ± 23.1	100.6 ± 24.3	0.019
C3, mg/dl	114,2 ± 25,9	106,3 ± 35,5	0.104	117.8 ± 19.5	113.4 ± 24.1	0.387	109.8 ± 31.5	102.2 ± 40.3	0.329
C4, mg/dl	23.7 ± 17.6	19.4 ± 7.5	0.065	23.3 ± 6.9	21.8 ± 7.0	0.351	24.1 ± 25.4	17.9 ± 7.5	0.141
CRP, mg/dl	1.0 ± 2.0	0.8 ± 1.4	0.516	0.7 ± 0.8	0.7 ± 1.0	0.778	1.4 ± 2.8	0.9 ± 1.6	0.295
median mRSS (range)	-	-	-	9 (43)	9 (40)	0.865	-	-	-
median SLEDAI (range)	-	-	-	-	-	-	8 (26)	10 (60)	0.813
median SLICC (range)	-	-	-	-	-	-	3 (7)	2 (8)	0.070
Autoantibodies:									
- ANA, current or former	101 (93.5%)	78 (95.1%)	0.760	56 (94.9%)	30 (96.8%)	1.000	45 (91.8%)	48 (94.1%)	0.712
- SSA	26 (24.1%)	18 (22%)	0.731	8 (13.6%)	2 (6.5%)	0.484	18 (36.7%)	16 (31.4%)	0.571
- SSB	7 (6.5%)	8 (9.8%)	0.407	1 (1.7%)	0 (0%)	1.000	6 (12.2%)	8 (15.7%)	0.620
- ssDNA	14 (13%)	22 (26.8%)	0.016	4 (6.8%)	3 (9.7%)	0.688	10 (20.4%)	19 (37.3%)	0.063
- dsDNA	14 (13%)	29 (35.4%)	< 0.001	3 (5.1%)	2 (6.5%)	1.000	11 (22.4%)	27 (52.9%)	0.002
- SM	3 (2.8%)	4 (4.9%)	0.467	1 (1.7%)	0 (0%)	1.000	2 (4.1%)	4 (7.8%)	0.678
- Nukleosomes	7 (6.5%)	15 (18.3%)	0.012	0 (0%)	0 (0%)	-	7 (14.3%)	15 (29.4%)	0.068
- RNP	6 (5.6%)	11 (13.4%)	0.060	2 (3.4%)	0 (0%)	0.543	4 (8.2%)	11 (21.6%)	0.092
- SCL70	12 (11.1%)	15 (18.3%)	0.160	12 (20.3%)	14 (45.2%)	0.014	0 (0%)	1 (2%)	1.000
- Centromere	29 (26.9%)	11 (13.4%)	0.024	28 (47.5%)	8 (25.8%)	0.046	1 (2%)	3 (5.9%)	0.618
- PMScl	2 (1.9%)	2 (2.4%)	1.000	1 (1.7%)	2 (6.5%)	0.272	1 (2%)	0 (0%)	0.490
- U1RNP	0 (0%)	1 (1.2%)	0.432	0	1 (3.2%)	0.244	0 (0%)	0 (0%)	-

- C1q	6 (5.6%)	6 (7.3%)	0.621	3 (5.1%)	2 (6.5%)	1.000	3 (6.1%)	4 (7.8%)	1.000
- RIB-PP	0 (0%)	3 (3.7%)	0.079	0 (0%)	0 (0%)	-	0 (0%)	3 (5.9%)	0.243
Antiphospholipid antibodies	10 (9.3%)	9 (11%)	0.696	3 (5.1%)	2 (6.5%)	1.000	7 (14.3%)	7 (13.7%)	1.000
- ß2-Glycoprotein I IgG	0%	4 (4.9%)	0.033	0 (0%)	2 (6.5%)	0.116	0 (0%)	2 (3.9%)	0.495
- ß2-Glycoprotein I IgM	7 (6.5%)	4 (4.9%)	0.639	3 (5.1%)	0 (0%)	0.549	4 (8.2%)	4 (7.8%)	1.000
- Cardiolipin IgG	1 (0.9%)	3 (3.7%)	0.317	0 (0%)	1 (3.2%)	0.244	1 (2.0%)	2 (3.9%)	1.000
- Cardiolipin IgM	3 (2.8%)	4 (4.9%)	0.467	2 (3.4%)	0 (0%)	0.543	1 (2.0%)	4 (7.8%)	0.363
- Lupus anticoagulans	3 (2.8%)	1 (1.2%)	0.635	0 (0%)	0 (0%)	-	3 (6.1%)	1 (2.0%)	0.357
corticosteroid use, current or former	57 (52.8%)	48 (58.5%)	.0.429	28 (47.5%)	15 (48.4%)	0.933	33 (64.7%)	29 (59.2%)	0.570
- 5-yr daily dose of prednisone, mg	1.9 ± 3.2	1.4 ± 2.6	0.324	2.1 ± 3.2	1.1 ± 2.4	0.100	1.6 ± 3.3	1.6 ± 2.7	0.885
- duration of corticosteroid use, months	31.8 ± 53.8	20.9 ± 49.0	0.157	37.2 ± 61.6	13.1 ± 29.8	0.016	25.1 ± 41.8	25.4 ± 57.0	0.978
- cumulative corticosteroid dose, g	7.8 ±15.6	4.0 ± 8.0	0.037	8.0 ± 14.8	2.7 ± 7.5	0.030	7.5 ± 16.9	4.8 ± 8.3	0.335
hydroxychloroquine use, current or former	26 (24.1%)	26 (31.7%)	0.242	5 (8.5%)	4 (12.9%)	0.489	21 (42.9%)	22 (43.1%)	0.977
- duration of hydroxychloroquine use, months	7.2 ± 26.3	12.9 ± 39.9	0.237	1.7 ± 10.4	1.0 ± 4.3	0.750	14.0 ± 36.6	20.1 ± 49.2	0.485
- cumulative hydroxychloroquine dose, g	59.9 ± 249.5	105.3 ±320.4	0.275	14.5 ± 93.8	11.5 ± 51.6	0.870	115.8 ± 351.1	162.4 ± 394.8	0.538
azathioprine use, current or former	32 (29.6%)	20 (24.4%)	0.422	14 (23.7%)	5 (16.1%)	0.588	18 (36.7%)	15 (29.4%)	0.436
- duration of azathioprine use, months	15.4 ± 40.1	7.8 ± 26.0	0.124	10.0 ± 29.6	7.9 ± 27.7	0.747	21.8 ± 50.5	7.7 ± 25.2	0.086
- cumulative azathioprine dose, g	54.3± 177.6	24.6 ± 80.4	0.127	29.4 ± 83.0	23.7 ± 83.0	0.763	83.9 ± 244.6	25.1 ± 79.6	0.115
cyclophosphamid use, current or former	10 (9.3%)	12 (14.6%)	0.251	2 (3.4%)	2 (6.5%)	0.606	8 (16.3%)	10 (19.6%)	0.669
- duration of cyclophosphamid use, months	1.5 ± 10.9	2.3 ± 11.9	0.620	0.2 ± 0.9	0.6±2.5	0.334	3.0 ± 16.1	3.2 ± 14.9	0.934
- cumulative cyclophosphamid dose, g	2.0 ± 16.0	1.0 ± 4.5	0.607	0.1 ± 0.6	0.8 ± 3.6	0.334	4.2 ± 23.6	1.2 ± 5.1	0.386

Values are number of patients (percentage) and mean +/- SD (if not other stated).
mRSS = modified Rodnan Skin Score, SLEDAI = Systemic Lupus Erythematosus Disease Activity Index, SLICC = Systemic Lupus International Collaborating Clinics damage index
