# Supplementary material for: Atherosclerotic plaques occur in absence of intima-media thickening in both systemic sclerosis and systemic lupus erythematosus: a duplexsonography study of carotid and femoral arteries and follow-up for cardiovascular events
Source: Arthritis Res Ther. 2014 Feb 19;16(1):R54. doi: 10.1186/ar4489 (PMC3978872; doi:10.1186/ar4489)
Supplement: Additional file 3 — Is tables presenting explorative analysis and additional multivariate Cox regression analysis of predictors of incident CVEs during follow-up in SSc and SLE patients. The first table shows a detailed report of explorative analysis of predictors of CVEs during follow-up in SSc and SLE patients. The second table shows additional multivariate Cox regression analysis of predictors of incident CVEs during follow-up in SSc and SLE patients including only carotid but not femoral artery plaques (if only carotid artery duplexsonography was performed and status of femoral artery plaque was unknown). [file ar4489-S3.pdf]

Additional file 3

Explorative analysis of potential predictors of cardiovascular events during follow-up in SSc and SLE patients

SSc and SLE (n = 129)			
Variable	beta coefficient	unadjusted hazard ratio (95% CI)	p-value
SSc vs SLE	-0.173	0.84 (0.33-2.10)	0.712
Age (per 5 years)	0.262	1.30 (1.10-1-54)	0.003
Male sex	2.833	17.00 (5.99-48.22)	<0.001
Carotid (CP) and femoral artery plaque (FP)			
- group 3 (CP & FP) vs group 1 (no plaque)	2,595	13.40 (2.99-59.95)	<0.001
- group 3 (CP & FP) vs group 2 (CP or FP)	2.672	14.47 (2.51-83.55)	0.003
- group 2 (CP or FP) vs group 1 (no plaque)	-0.077	0.93 (0.12-7.39)	0.942
Postmenopausal status, women only	1.576	4.83 (0.61-38.22)	0.135
Body-mass index (BMI)	0.063	1.07 (0.96-1.19)	0.253
Adipositas (BMI > 30)	0.517	1.68 (0.47-5.97)	0.425
Arterial hypertension	1.678	5.35 (1.91-15.02)	<0.001
Nicotine use (per 5 pack-years)	0.143	1.15 (1.04-1.28)	0.005
Pulmonary hypertension	0.124	1.13 (0.26-4.96)	0.869
Coronary heart disease	2.592	13.36 (4.67-38.20)	<0.001
Peripheral arterial vascular disease	0.983	2.67 (0.60-11.86)	0.196
Diabetes	-0.477	0.62 (0.29-1.32)	0.217
Dyslipidemia	0.484	1.16 (0.45-2.98)	0.766
Family history cardiovascular events	0.081	1.08 (0.31-3.80)	0.900
Age at diagnosis (per 5 years)	0.075	1.20 (1.04-1.39)	0.014
Disease duration (per 5 years)	0.109	1.11 (0.89-1.39)	0.334
GFR (Mayo, per 10 units)	-0.041	0.96 (0.77-1.20)	0.960
C3 (per mg/dl)	0.010	1.01 (0.99-1.03)	0.306
C4 (per mg/dl)	-0.027	0.97 (0.91-1-05)	0.474
CRP (per mg/dl)	0.220	1.25 (0.98-1.59)	0.079

CP = carotid artery plaque; FP = femoral artery plaque; GFR = glomerular filtration rate

Multivariate Cox regression analysis of predictors of incident cardiovascular events during follow-up in SSc and SLE patients (if only carotid artery duplexsonography was performed and status of femoral artery plaque would have been unknown)

Cox model (only carotid artery plaque) – SSc and SLE (n = 129)*			
Variable	beta coefficient	adjusted hazard ratio (95% CI)	p-value
Coronary heart disease	2.576	13.14 (3.96-43.64)	<0.001
Male sex	2.523	12.47 (3.96-39.27)	<0.001
Carotid plaque (yes vs no)	1.260	3.53 (0.97-12.87)	0.057

* 0 observations excluded from model because of missing values. Events per variable: 19/3 = 6.33
